# N-terminal peptide fragment constitutes core of amyloid deposition of serum amyloid A: An imaging mass spectrometry study

**DOI:** 10.1371/journal.pone.0275993

**Published:** 2022-10-14

**Authors:** Yukako Shintani-Domoto, Yuki Sugiura, Makiko Ogawa, Eiji Sugiyama, Hiroyuki Abe, Takashi Sakatani, Ryuji Ohashi, Tetsuo Ushiku, Masashi Fukayama

**Affiliations:** 1 Department of Pathology, Graduate School of Medicine, The University of Tokyo, Tokyo, Japan; 2 Department of Diagnostic Pathology, Nippon Medical School Hospital, Tokyo, Japan; 3 Department of Biochemistry, Keio University School of Medicine, Tokyo, Japan; 4 Department of Pathology, Memorial Sloan Kettering Cancer Center, New York, New York, United States of America; 5 Laboratory of Analytical and Bio-Analytical Chemistry, School of Pharmaceutical Sciences, University of Shizuoka, Shizuoka, Japan; 6 Department of Integrated Diagnostic Pathology, Nippon Medical School, Tokyo, Japan; 7 Asahi Tele Pathology Center, Asahi General Hospital, Asahi-City, Chiba, Japan; Consejo Superior de Investigaciones Cientificas, SPAIN

## Abstract

Serum amyloid A (SAA) is an acute phase protein, which undergoes structural changes and deposits in the extracellular matrix, causing organ damage. Systemic AA amyloidosis is a relatively common amyloid subtype among the more than 30 amyloid subtypes, but the mechanism of amyloid fibril formation remains unclear. In this study, we investigated the tissue distribution of SAA derived peptides in formalin-fixed paraffin embedded (FFPE) specimens of human myocardium with amyloidosis using matrix-assisted laser desorption/ionization imaging mass spectrometry (MALDI-IMS). In the whole SAA protein, four trypsin-digested peptides in the range of SAA2-67 were visualized and the N-terminal peptide; SAA2-15, was selectively localized in the Congo red-positive region. The C-terminal peptides; SAA47-62, SAA48-62, and SAA63-67 were detected not only in the Congo red-positive region but also in the surrounding negative region. Our results demonstrate that the N-terminal SAA2-15 plays a critical role in the formation of AA amyloid fibril, as previously reported. Roles of the C-terminal peptides require further investigation.

## Introduction

Amyloidosis is a disease in which amyloid precursor proteins form β-sheet structures that are deposited in the extracellular matrix, resulting in organ damage. In the diagnosis of amyloidosis, amyloid deposition is verified by Congo red staining, and the amyloid precursor protein is generally identified by immunohistochemistry [1]. To date, 36 amyloid precursor proteins have been reported [2]. In immunohistochemistry, it is often challenging to determine the type of amyloid due to false-positive or -negative immunoreactivity, or a suspected deposition of multiple amyloid precursor proteins. In these cases, liquid chromatography mass spectrometry (LC-MS/MS) is utilized to identify the type of amyloid species [1], and the absolute quantification of specific amyloid proteins using isotope labeling techniques has also become available [3]. Matrix-assisted laser desorption/ionization imaging mass spectrometry (MALDI-IMS) [4,5] has recently been used to identify and characterize amyloid precursor proteins directly on thin tissue sections, such as tissue microarrays. This new technique has been used to identify systemic amyloidosis [6–9] and amyloid-β deposits in the human brain [10,11]. In Alzheimer’s disease or cerebral amyloid angiopathy, the distribution of truncated C-terminal fragments of amyloid-β is altered in the brain tissue, as visualized by MALDI- Time Of Flight (TOF) MS [10,11].

Serum amyloid A (SAA)-related (AA) amyloidosis is most frequently observed in autopsy cases [3,12], despite the fact that light chain-related amyloidosis and transthyretin (TTR)-related (ATTR) amyloidosis are arguably more common than it had been previously estimated [13]. SAA is a representative member of a family of inflammatory acute-phase proteins, which are a group of proteins up or down-regulated during inflammation. SAA is upregulated in response to inflammation (e.g., in response to interleukin-6 and tumor necrosis factor) [14]. The incidence of AA amyloidosis has been decreasing given the successful treatment of chronic inflammatory diseases, such as use of biopharmaceuticals to control rheumatoid arthritis. Native SAA which displaying a unique four-helix bundle fold stabilized by its long C-terminal tail exists as a hexamer [15–17]. These reports clearly indicated that N-terminal fragments play a critical role in the formation of protein AA aggregates. However, it remains unclear how SAA peptides are truncated *in vivo* during the process of deposition as amyloid. Escape from endogenous clearance is necessary for the formation, aggregation, nucleation, and elongation of pathogenic amyloid fibrils, and such a mechanism has been proposed for proteolytic selection [18,19].

This study aimed to elucidate the core peptide by investigating the distribution of tryptic peptides of SAA using MALDI-IMS analysis. Concordant or discordant localization of peptides with amyloid deposition depends on the importance of each peptide in amyloid genesis. In this study, MALDI-IMS was applied to cardiac tissues of patients with AA amyloidosis, which were formalin-fixed and paraffin-embedded (FFPE). To evaluate the applicability of this method in the study of amyloid genesis, we also investigated associated proteins that are frequently observed in amyloid deposition, such as serum amyloid P (SAP), apolipoprotein A4 (Apo A4), apolipoprotein E (Apo E), and vitronectin (VTN).

## Materials and methods

### Ethics statement

The study protocol was performed according to the Declaration of Helsinki and was approved by the Research Ethics Committee of the Graduate School of Medicine, The University of Tokyo (No. 10461-4-(5)) and the Human Ethics Committee of Nippon Medical School (No. B-2020-200). Autopsies were performed with informed consent. Moreover, this study was conducted using autopsy records from the past, and we could not obtain informed consent from the bereaved family for the use of the records. Therefore, in accordance with the "Ethical Guidelines for Medical Research Involving Human Subjects (enacted by the Ministry of Health, Labor and Welfare in Japan), the opportunity for bereaved family to refuse was guaranteed by indicating the opt-out.

### Epitope mapping

To determinate the areas of activity/binding in a protein with a known amino acid sequence, a 1aa shifted sequence of the 15-mer peptide of pro SAA at 122aa was synthesized and probed with SAA mouse monoclonal antibody (mc1).

- SPOT Synthesis of the Arrays
  SPOT synthesis of 27 arrays was carried out using a MultiPep synthesizer (Intavis) [20]. Commercially available amino-PEG cellulose membranes were used in this study. The peptides were synthesized using solutions of pre-activated amino acids. Cleavage of the amino acid protecting groups was carried out using TFA solutions with scavengers (water, triisopropanol, and thioanisole). After cleavage, the membrane with the array was stored at -20°C until probing.
- Probing of the arrays
  First, as a negative control, the array was probed with a secondary antibody (peroxidase-labeled goat anti-mouse IgG2a antibody at a 1:30,000 dilution). The primary probing step was mimicked using T-TBS at the corresponding incubation time. The bound secondary antibody was detected using enhanced chemiluminescence (ECL). The ECL scan was performed at a scanning time of 300 s. Eight images of the scanned array were generated during that scanning time. Immediately after probing, two-step regeneration of the peptide arrays was carried out (without adding β-mercaptoethanol (BME)), and the membranes were stored at -20°C until probing with the desired protein.

Since there was no information about the specific concentration of the SAA antibody in the provided solution, the array was probed with SAA1 mc1 mouse monoclonal Ab at a dilution of 1:1000. Detection of bound protein was performed using peroxidase-labeled goat anti-mouse IgG2a antibody at a 1:30,000 dilution. The ECL scan was performed at a scanning time of 50 s with the generation of eight images during the scanning time.

### Patients and tissue samples for first cohort

Specimens from 30 patients with systemic amyloidosis were retrieved from the autopsy records of the Department of Pathology at the University of Tokyo Hospital (Tokyo, Japan) from January 1999 to December 2016. The diagnosis of systemic amyloidosis was based on the presence of amyloid in FFPE tissue sections from more than two organs, as detected by Congo red staining. Of these 30 patients, four were AA amyloidosis-affected patients and 2 ATTR patients, with moderate deposits detected by IHC [3], in addition to one patient without amyloidosis. Myocardial samples were FFPE tissue blocks from the left ventricle of the resected heart at autopsy in each case.

A tissue cylinder (2 mm in diameter) was placed in the recipient block for tissue microarray (TMA) analysis. Serial 3-μm-thick tissue sections of the TMA were used for hematoxylin and eosin (H&E) and Congo red staining, as well as SAA and TTR analysis using IHC. Additionally, 3-μm-thick serial sections were deposited onto indium tin oxide-coated glass slides (Bruker Daltonics Inc., Billerica, MA, USA) for IMS. For further analysis, the samples were deparaffinized by immersion in xylene at 60°C for 10 min twice and then washed twice in 100% ethanol for 5 min, followed by washing with 90, 80, and 70% aqueous ethanol solutions for 5 min each.

### Patients and tissue samples for second cohort

Of the myocardial specimens from 40 patients with systemic amyloidosis autopsied at the Department of Diagnostic Pathology, Nippon Medical School Hospital (Tokyo, Japan) between January 1982 and December 2020, five cases with AA amyloidosis were used as the second dataset (paper in progress). The diagnosis of systemic amyloidosis was the same as described above. For TMA analysis, tissue cylinder (3 mm in diameter) was collected and placed in the recipient block. Specimen preparation was performed in the same manner as described above for the first cohort.

### Immunohistochemistry

To identify amyloid proteins, antibodies against SAA and TTR were used: anti-SAA antibody (mouse monoclonal, mc1; Dako Denmark A/S, Glostrup, Denmark, dilution 1:5000) and anti-TTR antibody (rabbit monoclonal, EPR3219; Abcam PLC, Cambridge, UK, 1:200). Immunostaining was performed using BenchMark ULTRA immunohistochemistry and an in　situ hybridization system (Roche Diagnostics, Basel, Switzerland). Amyloid deposition in Congo red-stained tissue sections was observed using a tetramethyl rhodamine isothiocyanate (TRITC) filter with excitation and emission maxima of 545 and 605 nm, respectively, with a BZ-X710 all-in-one fluorescence microscope (Keyence Ltd., Osaka, Japan).

### Tissue preparation for IMS analyses

For both types of IMS analyses, after hydration, the sections were incubated in a humidified chamber at 55°C overnight. Trypsin solution was prepared by dissolving 70 μg of trypsin (Sigma-Aldrich, St. Louis, MO, USA) in 280 μL of 50 mM ammonium hydrogen carbonate, which was then applied to the sections using an airbrush (Procon Boy FWA Platinum 0.2-mm caliber airbrush, Mr. Hobby, Tokyo, Japan) to prepare a slide. The sections were incubated in a humidified chamber at 37°C for 8 h.

### Standard resolution IMS analysis by MALDI-TOF MS

The matrix solution consisted of 10 mg of α-cyano-4-hydroxycinnamic acid (Shimadzu Corp., Kyoto, Japan) in 1 mL of 50% acetonitrile containing 0.1% formic acid for analyses using a Q-Exactive Orbitrap mass spectrometer (Thermo Fisher Scientific, Tokyo, Japan), coupled with an atmospheric pressure scanning microprobe MALDI ion source (AP-SMALDI10, TransMIT GmbH, Giessen, Germany). The matrix solution consisted of 100 mg of 2,5-dihydroxybenzoic acid (Bruker Daltonics Inc.) in 1 mL of 70% aqueous methanol/0.1% trifluoroacetic acid (v/v) for analysis using a MALDI-TOF-type instrument (Ultraflex Xtreme; Bruker Daltonics Inc.). A thin matrix layer was applied to the surface of the plates using an airbrush and volume, as described above. A MALDI-TOF-type instrument equipped with an Nd:YAG laser with a 1000-Hz repetition rate was used for tissue section analyses. The experimental parameters for MALDI imaging were as follows: positive ion mode, 50 μm laser pitch, and 200 laser irradiations per pixel. In this experiment, the acceleration voltage was set to 25 kV and the laser power was set to 50% (Bruker’s notation). Signals enriched in the region of amyloid deposits were selected and verified in three independent experiments covering the range of *m/z* 600–3200. All data were acquired and visualized using the FlexImaging 4.1 software (Bruker Daltonics Inc.) and SCiLS 2020a/2022a (Bruker Daltonics) software. The ion images for each *m/z* 0.1 interval from *m/z* 600 to 3200 were reviewed in succession, and *m/z* signals specific to the region of amyloid deposit reproducibly were selected. For the orbitrap mass spectrometer, signals within a mass range between 600 and 2000 were acquired with a mass resolving power of 70,000 at m/z 200. The raster step size was set at 70 μm. Thereafter, the spectral data were transformed to image data and analyzed using ImageQuest 1.1.0 (Thermo Fisher Scientific) software [21].

Protein-specific m/z values were selected, and their ion images were acquired by referring to the Orbitrap results, LC-MS/MS results of a case, and the MS-Digest database (For details, see Materials and methods in Reference [3]). Tables 1 and S1 show the list of tryptic peptides detected: four tryptic peptides from SAA and one tryptic peptide from TTR were detected, all of which were consistent with amyloid deposition sites in AA and ATTR cases. In addition, one SAP, one Apo A4, two Apo E, and seven VTN tryptic peptides were detected, consistent with the amyloid deposition site and its surroundings.

**Table 1:** Identification of peptides in cardiac tissues of patients with AA and ATTR amyloidosis.

Amyloid type	*m/z*	*m/z*	m/z*	*m/z* based on MS-Digest		
	IMS TOF/TOF	IMS Orbitrap	LC-MS Orbitrap	Mi	av	Ref.
SAA	659.377	659.347	N.D.	659.347	659.724	
SAA	1456.821	1456.725	1456.721	1456.718	1457.595	[7]
SAA	1550.823	1550.738	1550.725	1550.727	1551.709	
SAA	1612.934	1612.830	1612.821	1612.819	1613.780	[6–8]
TTR	1366.795	1366.772	N.D.	1366.759	1367.603	[6,8]
SAP	764.480	764.445	764.445	764.445	764.951	[6,8]
Apo A4	983.588	983.557	983.554	983.552	984.147	
Apo E	1033.632	1033.550	1033.546	1033.540	1034.170	[7]
VTN	1422.744	1422.650	1422.655	1422.654	1423.531	[6–8]

The m/z detected by IMS TOF/TOF was confirmed by IMS orbitrap, LC-MS/MS, and MS-Digest (http://prospector.ucsf.edu/prospector/cgi-bin/msform.cgi?form=msdigest).

* LC-MS/MS data were obtained from a case of AA amyloidosis (see related data in reference ^3^; http://www.peptideatlas.org/PASS/PASS01558).

IMS, imaging mass spectrometry; LC-MS, liquid chromatography mass spectrometry; mi, monoisotopic molecular mass; av, average molecular mass; Ref, reference; SAA, serum amyloid A; TTR, transthyretin; SAP, serum amyloid P component; Apo A4, apolipoprotein A4; ApoE, apolipoprotein E; VTN, vitronectin.

## Results

### The epitope of SAA (mc1) antibody utilized for clinical diagnosis is N-terminal sequence of SAA

**Fig 1:**
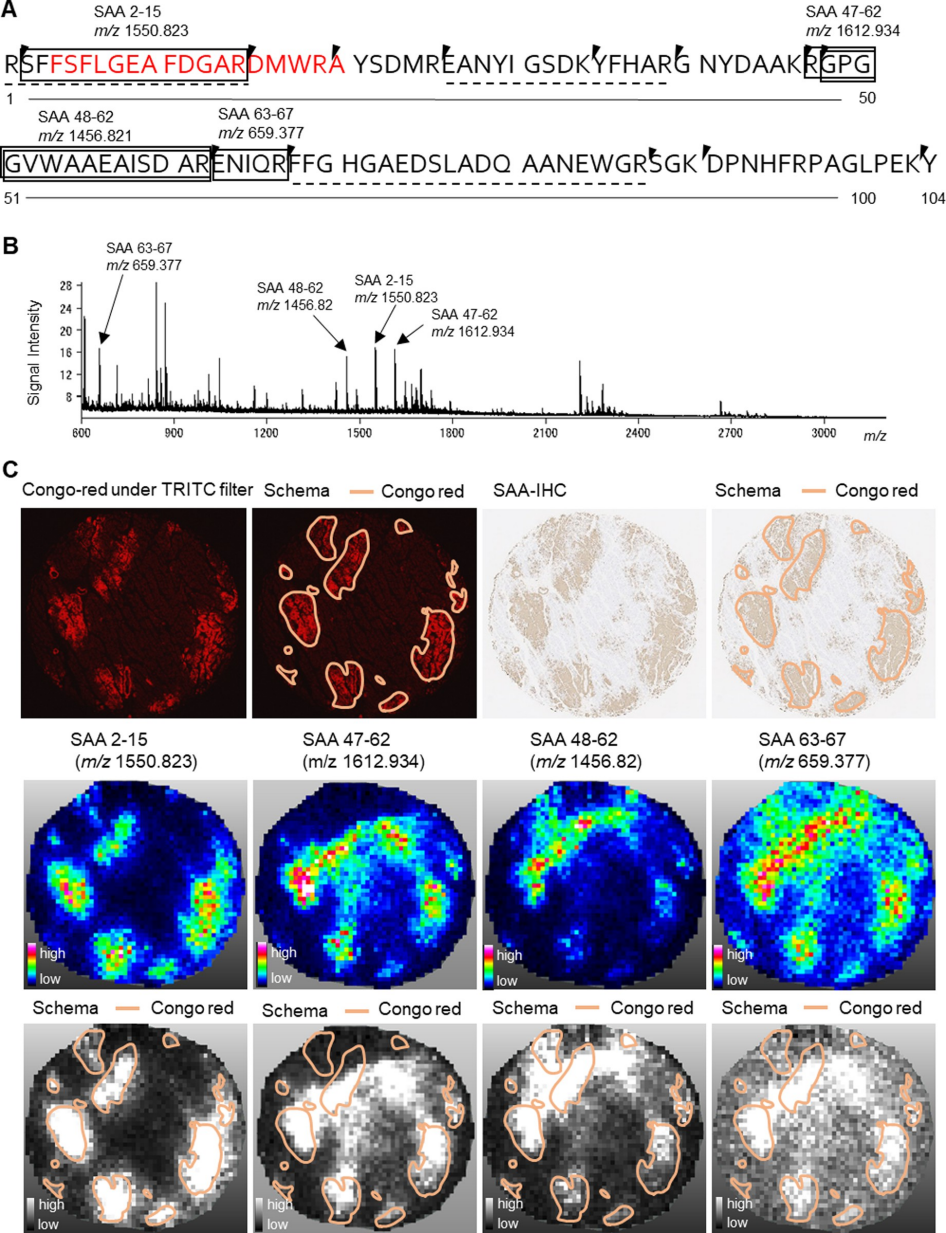
N-terminal peptides of serum amyloid A in cardiac tissues of AA amyloidosis. A. Sequence of human Serum Amyloid A1 (SAA1): SAA 4–20; shown in red, is the recognition site of antibody (mc1) to SAA. Four tryptic peptides, SAA 2–15, SAA 47–62, SAA 48–62, and SAA 63–67, detected by matrix-assisted laser desorption/ionization -time of flight spectrometry (MALDI-TOF MS) in this study, are enclosed in the box. Arrowheads indicate digestion sites by trypsin. Three tryptic peptides, SAA 1–15, SAA26-39 and SAA 68–87 with dotted lines were detected by liquid chromatography mass spectrometry (MS)/MS in the myocardium containing both amyloid deposits and background in previous report [3]. B. Averaged mass spectrum obtained from Cong-red positive area in the cardiac wall of Case 1 (AA amyloidosis). Note that four ion peaks (arrows) match the tryptic peptides of SAA 2–15 (*m/z* 1550.823), SAA 47–62 (*m/z* 1612.934), SAA 48–62 (*m/z* 1456.821), and SAA 63–67 (*m/z* 659.377). C. Distribution of signals in ion images of the cardiac wall of Case 1 (AA amyloidosis), as detected by MALDI-IMS. Top panels show the distribution of Congo red-positive staining and SAA immunoreactive in the cardiac wall of Case 1 (AA amyloidosis). From left to right: Amyloid deposits in red (Congo red staining) under tetramethyl rhodamine isothiocyanate (TRITC filter) on fluorescent microscopy with or without their tracing images, and SAA-IHC with or without their tracing images. Middle panels show ion images of the serial sections (left to right: SAA 2–15, SAA 47–62, SAA 48–62, and SAA 63–67). Bottom panels consist of ion images of the same sections with gray scale, superimposed by tracing of Congo red positive regions. Core diameter of histologic section, 2 mm. The resolving power (FWHM) measured from the average spectrum were ranged from 10000 to 13000 at m/z 1550.823.

Anti-SAA (mc1) antibody has been widely used in Japan to identify clinical amyloid typing [19,22,23], although that the information on the site of the epitope is not available. To examine the recognition region of the mc1antibody, partial SAA peptide sequences were probed with the mc1 antibody, yielding a distinct single binding region (S1 Fig). This binding region ranged from position A20 to position B1 (SFFSFLGEAFDGARDMWRAYSDMR), and the strongest binding appeared to occur in the region from position A22 to position A24 (FSFLGEAFDGARDMWRA), which is the amino acid sequence of SAA 4–20 on the N-terminal side of SAA (Figs 1A and S1).

### Different peptide regions of SAA showed different distribution patterns in the myocardium of patient with AA amyloidosis

To evaluate the distribution of peptides of SAA by MALDI-TOF MS, we selected a single index case in which a large amount of amyloid was deposited (Case 1 in Table 2). In this study, four tryptic peptides, SAA 2–15, SAA 47–62, SAA 48–62, and SAA 63–67, were detected in SAA 1–104 by MALDI-TOF MS after trypsin digestion (Fig 1A–1C). In the myocardium of a patient with AA amyloidosis, six tryptic peptides of SAA were detected by LC-MS in samples containing both amyloid deposits and background tissue: two overlapping pairs of SAA peptides ([1–15 and 2–15] and [47–62 and 48–62]), as well as SAA 26–39 and SAA 68–87 peptides (Fig 1B). One of the tryptic peptides detected by MALDI-IMS, SAA 63–67, was below the LC-MS detection level because of its short length (Table 1).

**Table 2:** Demographic features of patients with AA and ATTR amyloidosis and those without amyloidosis.

Case No.	Age	Gender	Amyloid type	Clinical diagnosis at autopsy	Year of Autopsy
1	49	F	AA	Rheumatoid arthritis	2003
2	64	M	AA	Rheumatoid arthritis	2005
3	71	F	AA	Systemic lupus erythematosus, Sjogren syndrome	2013
4	48	M	AA	Dilated cardiomyopathy	2011
5	77	F	ATTR	Senile systemic amyloidosis	2014
6	65	M	ATTR	Familial amyloidosis	2014
7	68	M	Normal	Esophageal carcinoma, hepatic carcinoma, and cirrhosis	2013

The distribution of SAA-IHC-positive regions, that is, the distribution of immunoreactivity to the SAA-N-terminal peptide, was almost the same as the distribution of Congo red-positive regions (Fig 1C, upper panels). In a few cases, due to differences in staining sensitivity, SAA-IHC may be positive, even in Congo red-negative regions [24]. Next, the ion images of each peptide were compared to the amyloid distribution (Fig 1C, middle and lower panels). The signals of SAA 2–15, the N-terminus of SAA, were localized in regions corresponding to the Congo red-positive region, while SAA 47–62, SAA 48–62, and SAA 63–67 were widely distributed beyond the Congo red-positive regions. Furthermore, there were several regions of amyloid deposition in which SAA 47–62 and SAA 48–62 showed no signal.

### First cohort; N-terminal tryptic peptide 2–15 of SAA selectively localized to the positive region of Congo red

**Fig 2:**
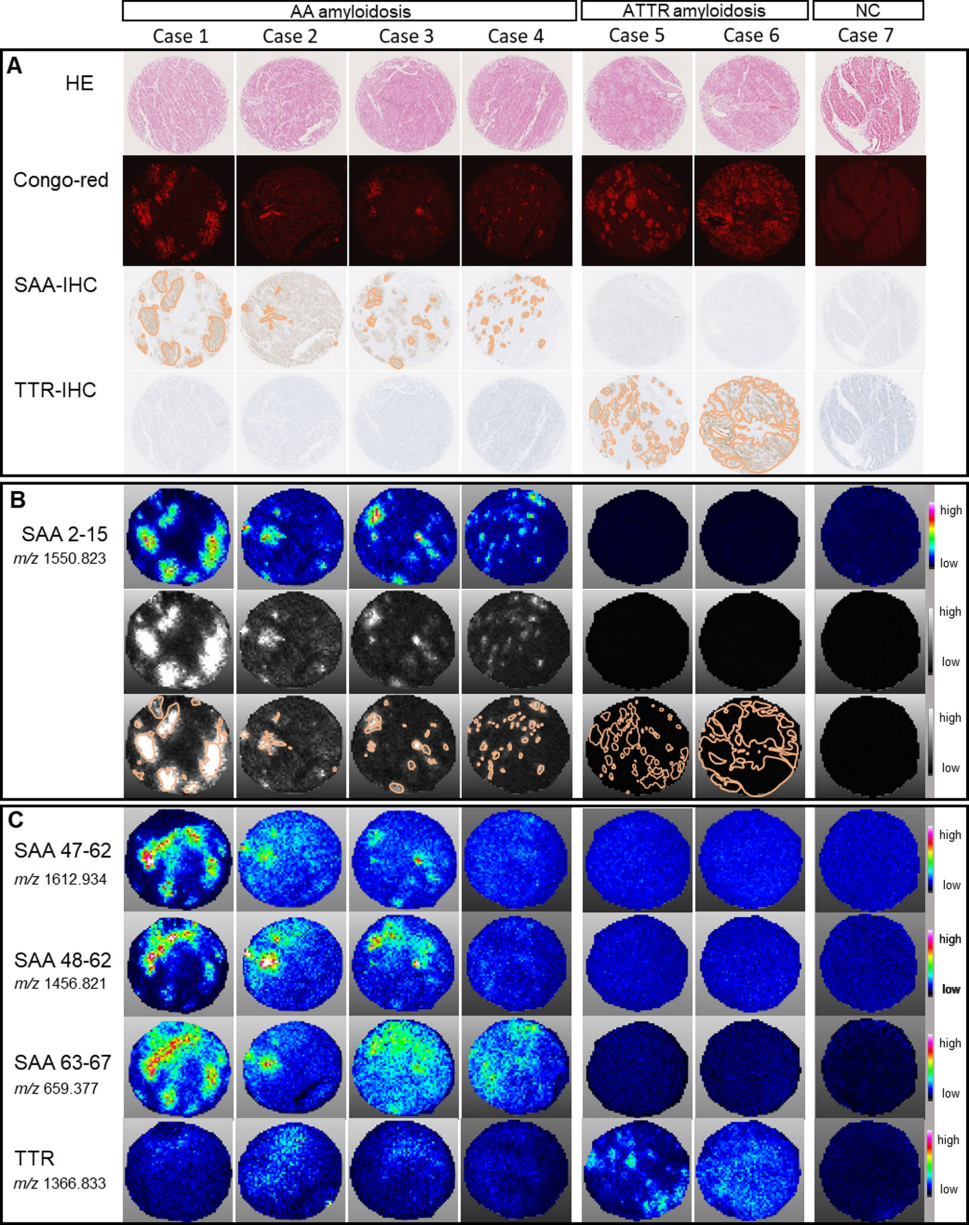
Serum Amyloid A (SAA) 2–15 is specifically localized to amyloid deposits in cardiac tissue of AA amyloidosis. A. From the upper panel, hematoxylin and eosin staining, Congo red staining, and immunohistochemistry (IHC) of SAA and transthyretin (TTR) were superimposed by tracing of Congo red-positive regions. B. Ion images of SAA 2–15 (*m/z* 1550.823) in AA amyloidosis (Case 1–4), ATTR amyloidosis (Cases 5 and 6), and normal control (Case 7). The three panels consist of color displays of ion images (upper), gray scale display (middle), and gray scale display superimposed by tracing of Congo red-positive regions (lower). The intensity range is given for each ion image on the right-hand side. The core diameter of the histologic section was 2 mm. C. Ion images of SAA 47–62 (*m/z* 1612.934), 48–62 (*m/z* 1456.821), SAA 63–67 (*m/z* 659.377) and transthyretin (TTR) (*m/z* 1366.833) in AA amyloidosis (Case 1–4), ATTR amyloidosis (Cases 5 and 6), and normal control (Case 7). The intensity range is given for each ion image on the right-hand side. The core diameter of the histologic section was 2 mm.

To further verify the above findings (observed in the index case), three additional cases of AA amyloidosis were examined using MALDI-IMS in comparison to patients with ATTR amyloidosis and normal controls without amyloidosis (Table 2 and Fig 2). Consistent with previous results, the ion images of SAA 2–15, the N-terminal of SAA, corresponded to the Congo red-positive region in Cases 2, 3, and 4 with AA amyloidosis, as in Case 1 (Fig 2B). No SAA 2–15 signal was observed in the ATTR cases or normal controls.

We then tested whether the signal distribution in SAA 47–62, SAA 48–62, and SAA 63–67, was more diffuse compared to SAA 2–15, and the results were still consistent with the index case (Fig 2C). The spread of the amyloid deposition signal was more pronounced in SAA 47–62 in case 2, SAA 48–62 in cases 2 and 3, and SAA 63–67 in cases 1, 3, and 4. In ATTR amyloidosis cases 5 and 6, no signals of SAA 47–62, SAA 48–62, and SAA 63–67 were found. In contrast, the localization of TTR-derived peptides corresponded to the Congo red-positive region of ATTR amyloidosis. The signals of TTR were weakly detected but the peaks were not detected in cases 1, 2 and 3 with AA amyloidosis, therefore no significant signal of TTR was detected in cases of AA amyloidosis.

### Second cohort; validation cohort obtained using data sets from different institutions

**Fig 3:**
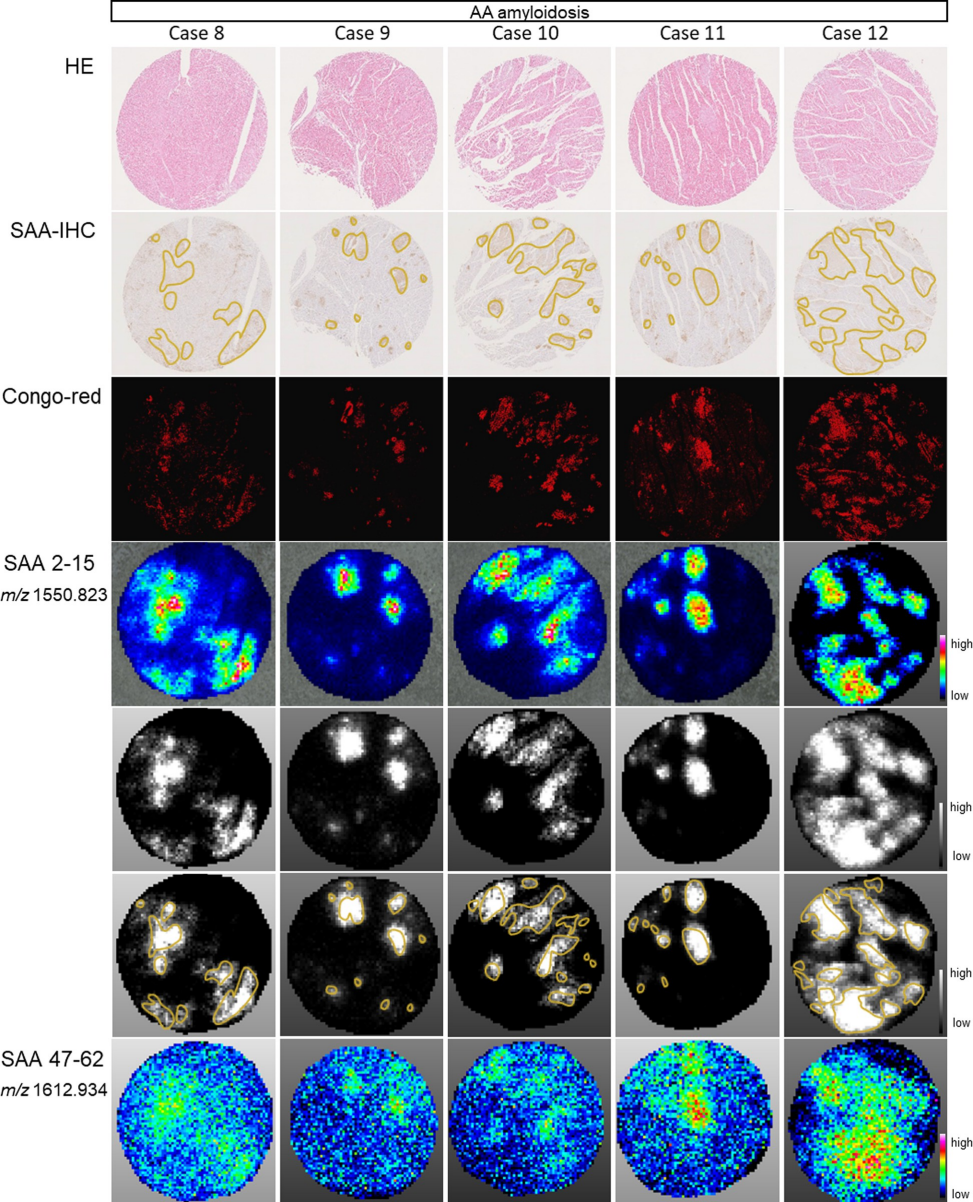
SAA 2–15 specifically localized to amyloid deposits in validation cohort from different institutions. From the upper, hematoxylin and eosin (H&E) staining, immunohistochemistry (IHC) of serum amyloid A (SAA), Congo red staining, and ion images acquired by MALDI-IMS using the second data set were superimposed by tracing of Congo red-positive regions, as needed. SAA 2–15 positivity was observed in Congo red-positive region in five cases with AA amyloidosis. In contrast, the signal of SAA 47–62 was weak and widely scattered in the surrounding area of Congo red-positive region. The core diameter of the histologic section was 3 mm.

To validate the results obtained in the first cohort, we performed validation using a dataset obtained from a different medical institution. Five cases of AA amyloidosis autopsied at Nippon Medical School Hospital were examined by MALDI-IMS (Table 3). Similar conclusions were drawn in this study. Comparing the ion images of SAA 2–15 (N-terminal peptide) and SAA 47–62, the signal of SAA 2–15 was consistent with the Congo red-positive region. In contrast, the signal of SAA 47–62 was widely scattered in the surrounding regions other than the Congo red-positive region (Fig 3).

**Table 3:** Demographic features of patients with AA amyloidosis in another dataset obtained from different institutions.

Case No.	Age	Gender	Amyloid type	Clinical diagnosis at autopsy	Year of Autopsy
8	65	F	AA	Rheumatoid arthritis	2006
9	58	F	AA	Rheumatoid arthritis	2000
10	64	F	AA	Rheumatoid arthritis	1999
11	71	F	AA	Rheumatoid arthritis	1992
12	57	M	AA	Rheumatoid arthritis	1983

### Identification of co-deposited proteins in human hearts with AA amyloidosis

**Fig 4:**
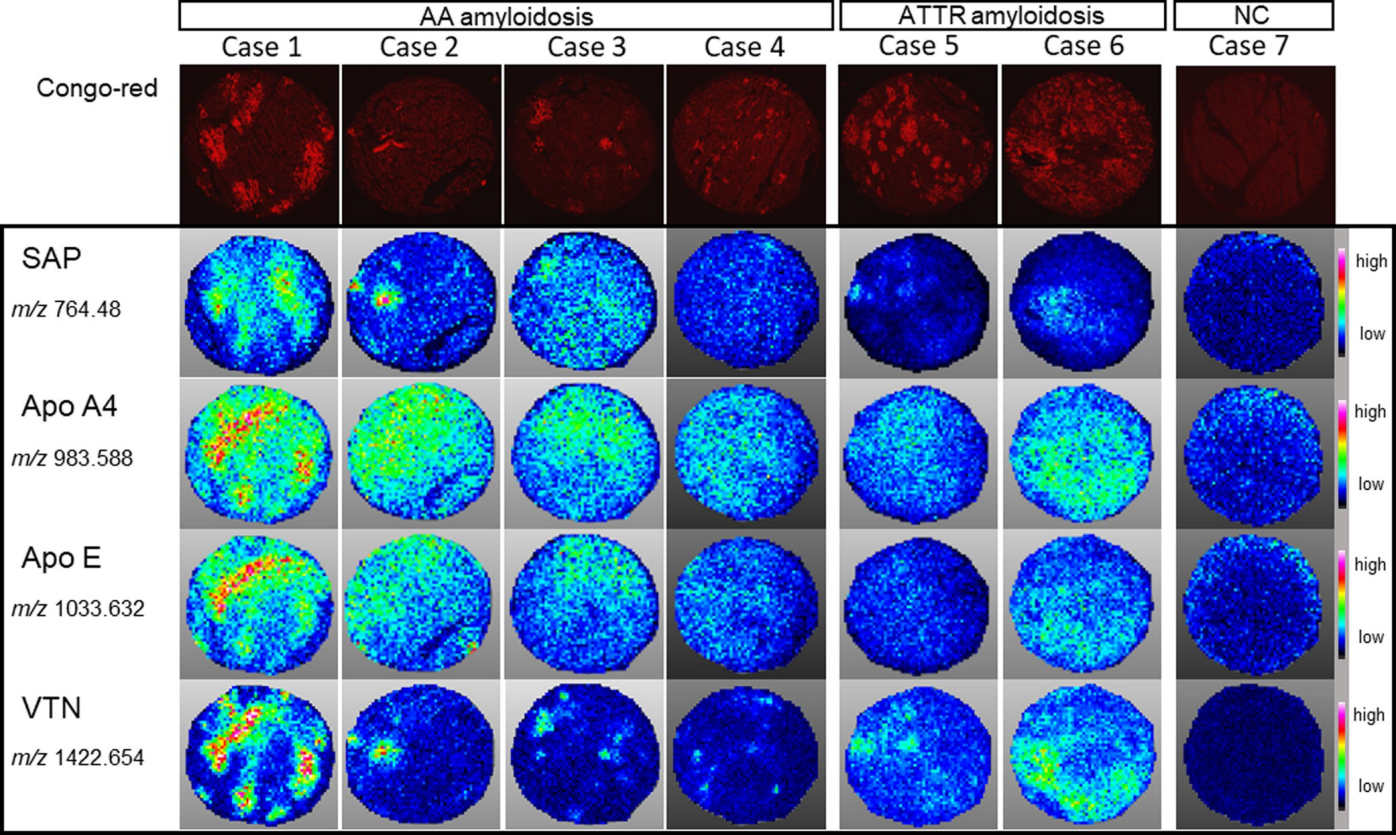
Ion images of co-deposited proteins in cardiac tissue of patients with AA and ATTR amyloidosis. Congo red staining (upper panels) and ion images of serum amyloid A (SAP) (*m/z* 764.48), apolipoprotein A4 (Apo A4) (*m/z* 983.588), apolipoprotein E (Apo E) (*m/z* 1033.632), and vitronectin (VTN) (*m/z* 1422.654) (lower panels). The intensity range is given for each ion image on the right-hand side. The core diameter of the histological sections was 2 mm.

MALDI-IMS was used to evaluate the co-deposition of amyloid-related proteins with SAA such as SAP, Apo A4, Apo E, and VTN (Fig 4). Among these co-deposited proteins, the tryptic peptides of VTN showed the same distribution as the Congo red-positive region in all four cases of AA amyloidosis and two cases of ATTR amyloidosis (Fig 4). In the present study, six additional peptides of VTN (m/z 875.517, 887.556, 1158.604, 1314.829, 1646.893, and 1666.908) were evaluated (S1 Table). These generally showed the same distribution pattern, although slight variations were observed (S2 Fig). However, partial correspondence was observed between Cases 1 and 2 for SAP, and between Cases 5 and 6 for Apo A4 and Apo E (Fig 4).

## Discussion

This study showed that SAA-N-terminal peptides selectively accumulate at Congo red-positive sites, which are recognized as amyloid deposition plaques. In particular, the SAA antibody (mc1), which is widely used for amyloid typing in Japan, recognized SAA 4–20, suggesting that accumulation of the N-terminal region is the basis for clinical diagnosis. This is consistent with the fact that SAA 2–15 were selectively detected in the Congo red-positive region by MALDI-IMS. Epitopes of mc1 were described in SAA 5–16 [25] and SAA 7–15 [26], which showed sites similar to our results.

It has been shown that N-terminus plays a critical role in amyloid formation, and the N-terminal side of SAA 1–11 in particular has been reported to be high-density lipoprotein (HDL)-binding site and important for amyloid fibril formation [27]. The N-terminal side is rich in phenylalanine and highly hydrophobic, and thus may be prone to amyloid fibril aggregation [28,29].

We detected four tryptic peptides in the range of 2–67 of SAA by MALDI-IMS, but no tryptic peptides in the C-terminal site. In agreement with our findings, Yamada et al. reported that the C-terminus of SAA is absent in human AA deposits via immunohistochemistry [30]. A recent study by Liberta et al. demonstrated that the most prominent cleavage site is located at residues 64–67 in four SAA patients [31]. Furthermore, a study using electron cryo-microscopy revealed that SAA 2–47 and SAA 2–64 were main constituents of extracted human amyloid fibrils (27). In our previous study, amyloid deposits from Congo red-stained slides were collected by laser microdissection and absolutely quantified tryptic peptides; SAA 26–34 and SAA 91–103 by LC-MS [3]. While SAA 26–34 were detected in all 42 AA samples, SAA 91–103 were found in 16 AA samples, illustrating that the average SAA 26-34/ SAA91-103 ratio was only 5.3% (0.4–9.9%) among these16 samples (Supplementary Data 3 Table of reference (3)). This indicates that the C-terminus is almost absent or may be present only in very small amounts in the amyloid deposits.

We found the distribution of the three C-terminal tryptic peptides; SAA47-62, SAA 48–62, and SAA 63–67 was different from N-terminal tryptic peptide; SAA 2–15 by MALDI-IMS. Further, we observed C-terminal tryptic peptides in the regions where both Congo red- and SAA-IHC were negative, in addition to the Congo red-positive area. We could not exclude the possibility that these three tryptic peptides distributed in the Congo red-negative region might be digested by endogenous proteases and be present without forming amyloid fibril (S3 Fig). On the other hand, the N-terminal side is located in the center of the amyloid fibers and may be resistant to the digestion [29]. Endogenous proteases, such as matrix metalloproteinases and cathepsins, could be produced by inflammatory cells or cardiomyocytes to cleave SAA proteins (S4 Fig).

SAA synergizes with chemokines to increase the chemoattraction between monocytes and granulocytes at the inflammatory site. SAA1 is cleaved by MMP9 and its C-terminal fragments cooperate with CXCL8 to activate and migrate neutrophils [32]. Cathepsins, which are endosomal and lysosomal proteases, have also been shown to cleave SAA [33]. Cathepsin B cleaves SAA at residues 76–77 to produce the most common form of AA found in amyloidosis [34,35]. Cathepsin D, a lysosomal enzyme, plays a role in truncating SAA at the N-terminus [36,37].

MALDI-IMS has been applied to various types of amyloid deposits, which has demonstrated co-deposition of VTN and apolipoproteins [7–9]. In this study, VTN-derived peptides were detected in all AA and ATTR amyloidosis cases, and their localization corresponded to amyloid deposition. Winter et al. demonstrated via immunohistochemistry that VTN is present in > 90% of the amyloid area in 85% of AA amyloidosis cases and 54% of ATTR amyloidosis [9]. This contrasts with the partial correspondence of SAP in AA amyloidosis and Apo A4 and Apo E in ATTR amyloidosis in our study, indicating the importance of VTN as an extracellular component involved in amyloid pathology.

This study has several limitations, first being the small number of cases. We included four cases in the first cohort of AA amyloidosis and five cases in the second cohort; however, the number is not sufficient. Second, there seem to be some differences in the peptides that are likely to be detected by the combination of MALDI-IMS and on-tissue digestion depending on how long the FFPE specimens are stored and how they have been fixed. In the first cohort, four peptides were detected by MALDI-IMS, whereas in the second cohort, two peptides were detected by MALDI-IMS. This may also be since the first cohort used autopsies from up to 19 years ago, whereas the second cohort used autopsies from 15 to 39 years ago. In future study, it will be necessary to analyze specimens from multiple medical institutions to handle a larger number of cases.

To conclude, we found that the SAA-N-terminal peptide selectively accumulates at the Congo red positive site more than at the C-terminal region. The fact that accumulation of the N-terminal region is the basis for clinical diagnosis by IHC, together with the fact that the N-terminal region is highly hydrophobic, leads to the suggestion that the N-terminal peptide plays a more important role in the formation of amyloid deposition plaques, a hypothesis that deserves further testing.

## Supporting information

S1 Fig The N-terminal sequence of SAA is the epitope recognized by mc1 antibody used for clinical diagnosis. Antibody to serum amyloid A (SAA) (mc1) binds the region spanning from position A20 to B1 (SFFSFLGEAFDGARDMWRAYSDMR), whereas the strongest binding occurs in the region spanning A22 to A24 (FSFLGEAFDGARDMWRA) located within SAA 4–20 sequence on the N-terminal.
(TIF)

S2 Fig Ion images of apoprotein E and vitronectin in myocardium of AA and ATTR amyloidosis. Congo red staining (upper panels) and ion images of Apoprotein E (Apo E) (m/z 1730.944) and vitronectin (VTN) (m/z 875.517, 887.556, 1158.604, 1314.829, 1646.893, 1666.908) (lower panels). The intensity range is given for each image on the right-hand side. The core diameter of the histologic section was 2 mm.
(TIF)

S3 Fig Schematic representation of the proteolysis in cardiac tissue of patients with AA amyloidosis. C-terminal side peptides, SAA47-62, SAA 48–62, and SAA 63–67 may be degraded by endogenous proteases and spread to the surrounding tissues.
(TIF)

S4 Fig SAA harbors cleavage sites for cathepsins and matrix metalloproteinases. Four regions, SAA 2–15, SAA47-62, SAA 48–62, and SAA 63–67 as detected by MALDI-TOF MS in this study, are shown in the box.
(TIF)

S1 Table Identification of peptides in cardiac tissues of patients with AA and ATTR amyloidosis. (DOCX)
