# Supplementary figures and images for: N-terminal peptide fragment constitutes core of amyloid deposition of serum amyloid A: An imaging mass spectrometry study
Source: PLoS One. 2022 Oct 14;17(10):e0275993. doi: 10.1371/journal.pone.0275993 (PMC9565386; doi:10.1371/journal.pone.0275993)

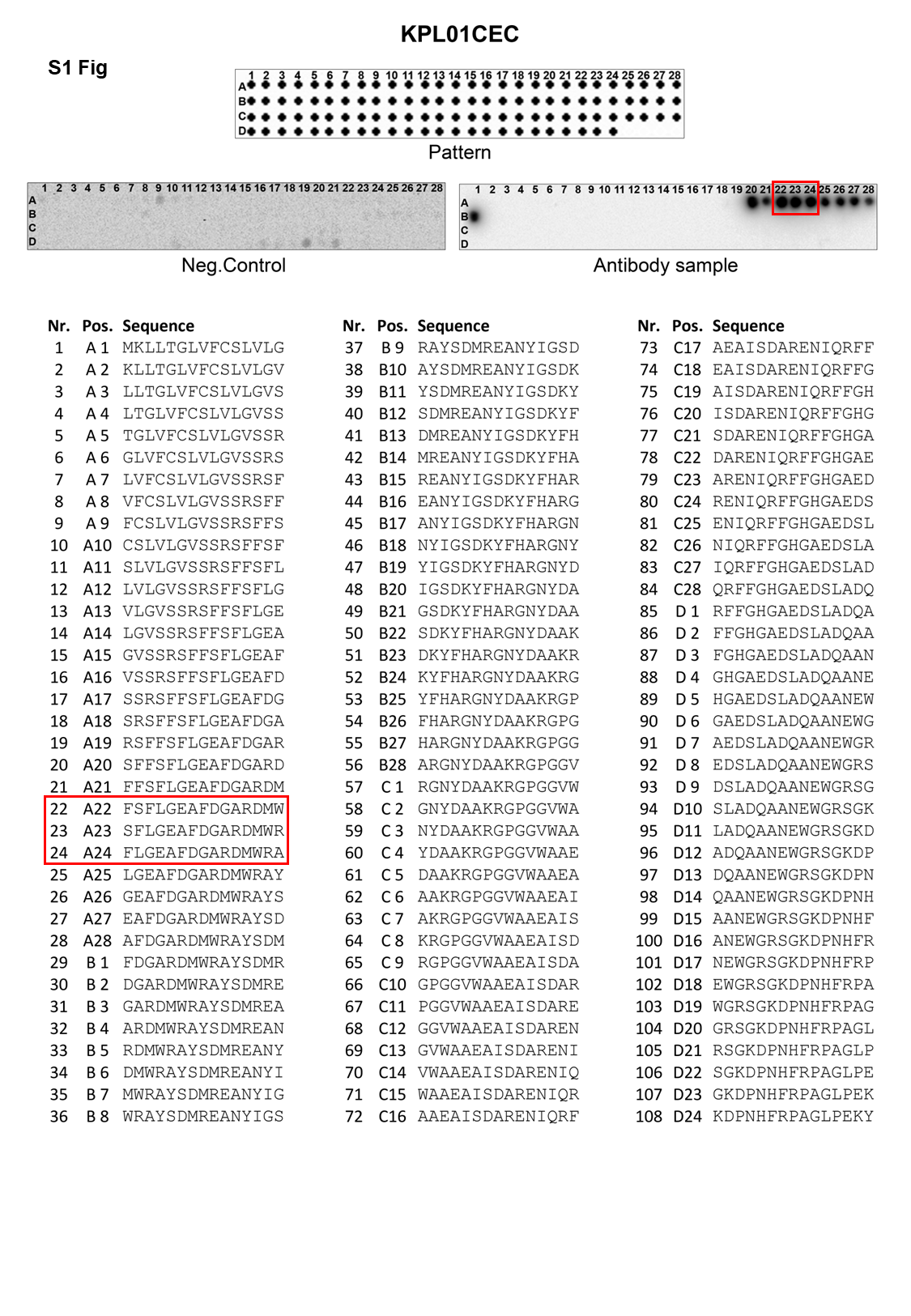

Supplement: S1 Fig — Antibody to serum amyloid A (SAA) (mc1) binds the region spanning from position A20 to B1 (SFFSFLGEAFDGARDMWRAYSDMR), whereas the strongest binding occurs in the region spanning A22 to A24 (FSFLGEAFDGARDMWRA) located within SAA 4–20 sequence on the N-terminal. (TIF) [file pone.0275993.s001.TIF]

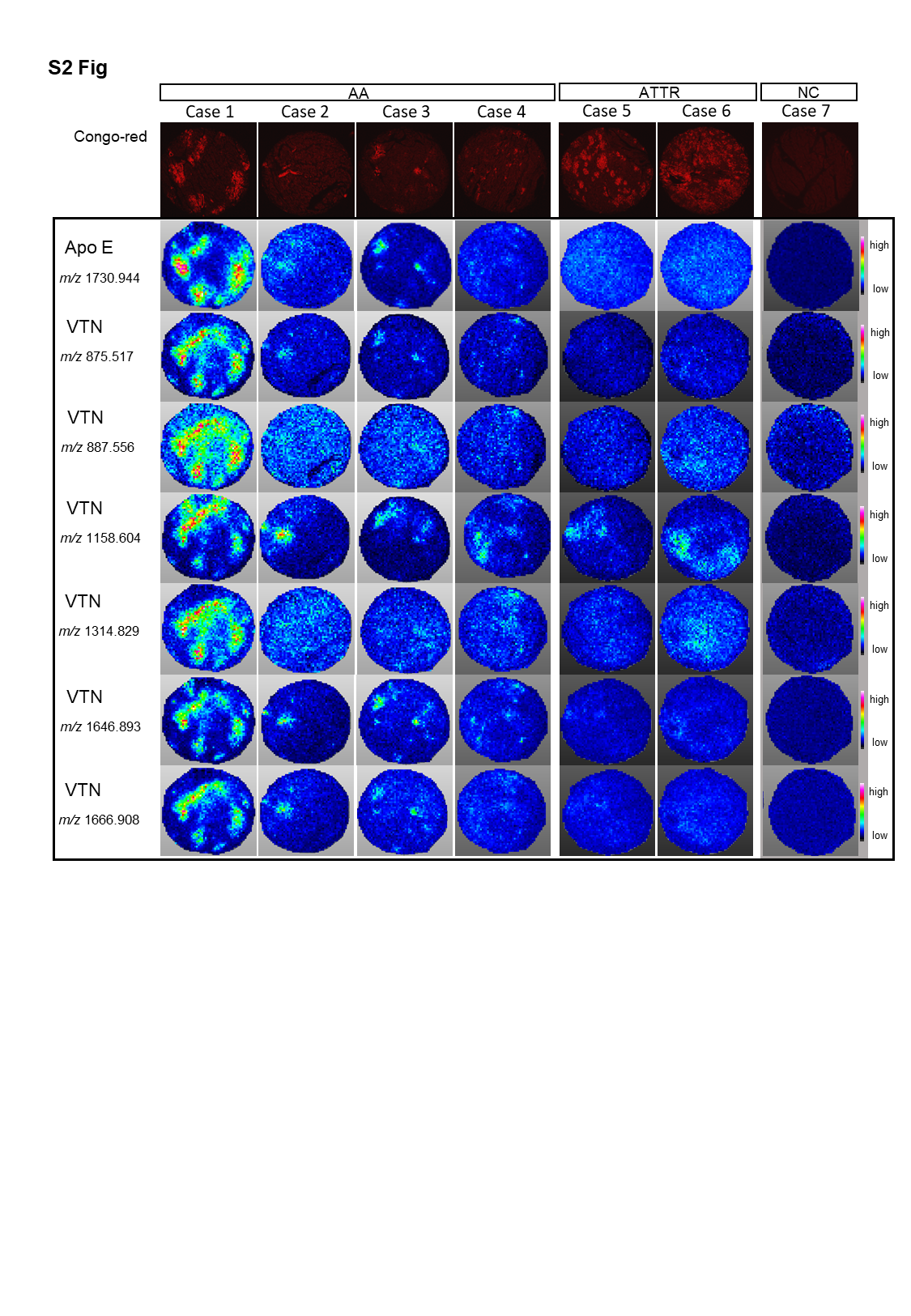

Supplement: S2 Fig — Congo red staining (upper panels) and ion images of Apoprotein E (Apo E) (m/z 1730.944) and vitronectin (VTN) (m/z 875.517, 887.556, 1158.604, 1314.829, 1646.893, 1666.908) (lower panels). The intensity range is given for each image on the right-hand side. The core diameter of the histologic section was 2 mm. (TIF) [file pone.0275993.s002.TIF]

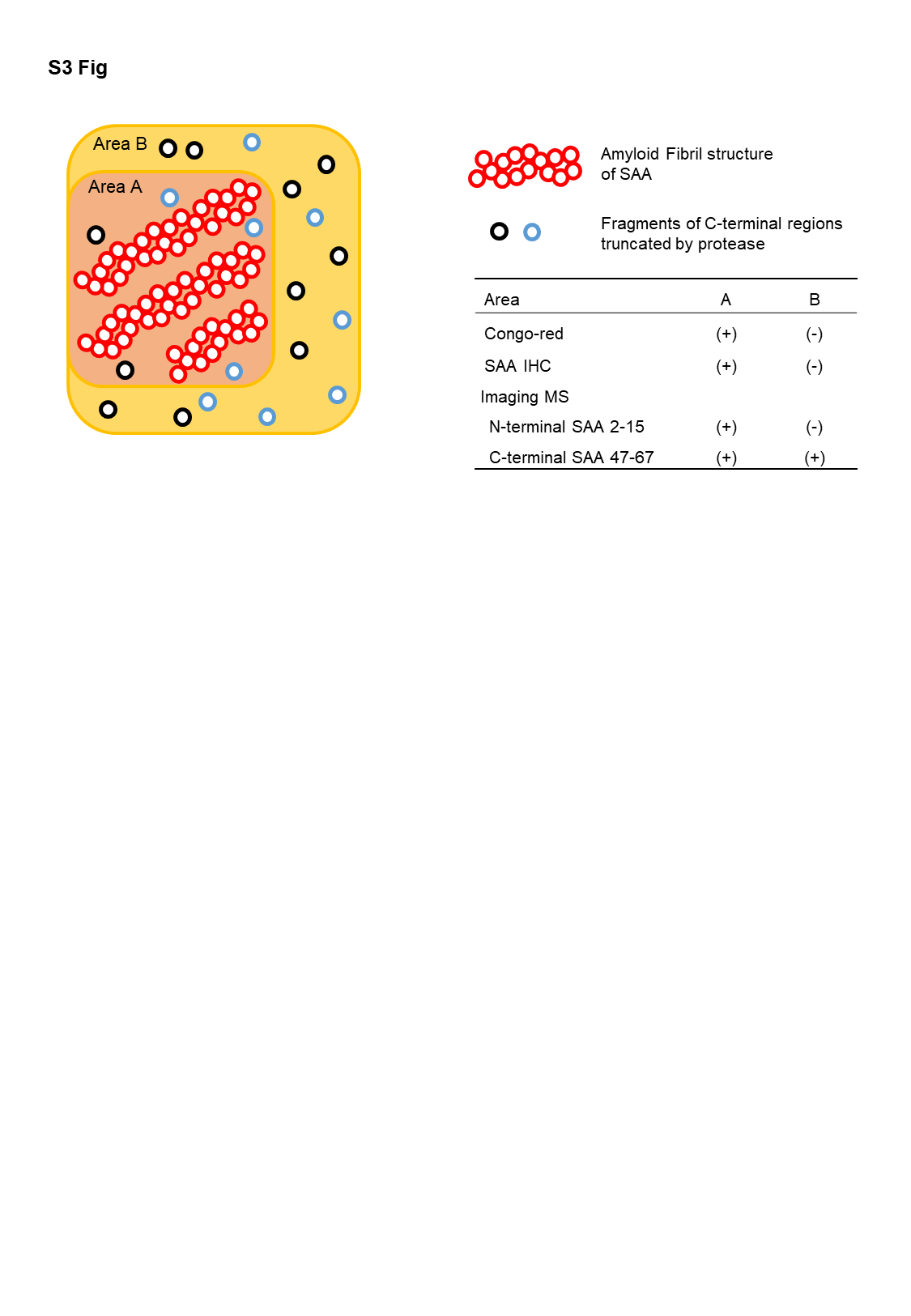

Supplement: S3 Fig — C-terminal side peptides, SAA47-62, SAA 48–62, and SAA 63–67 may be degraded by endogenous proteases and spread to the surrounding tissues. (TIF) [file pone.0275993.s003.TIF]

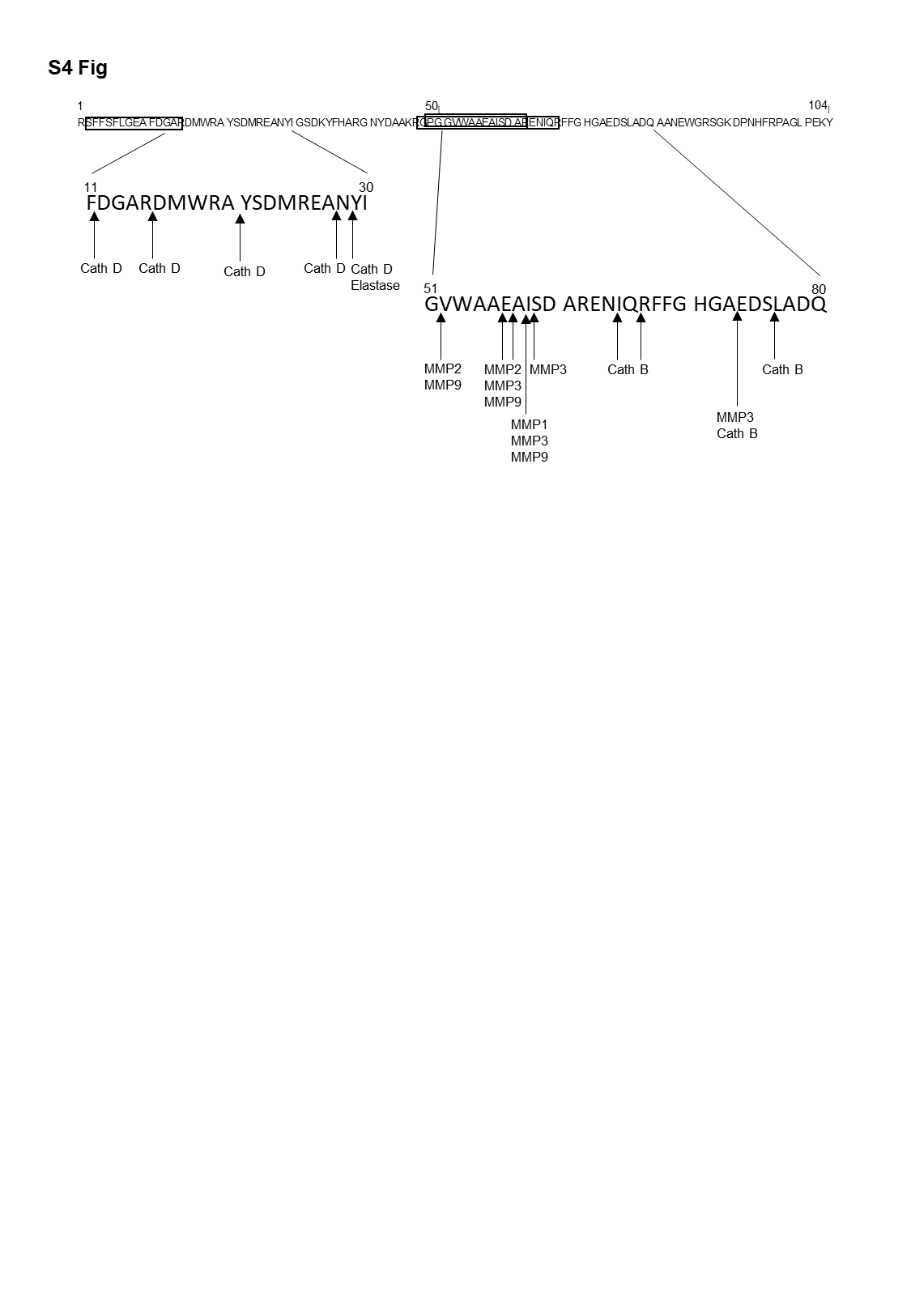

Supplement: S4 Fig — Four regions, SAA 2–15, SAA47-62, SAA 48–62, and SAA 63–67 as detected by MALDI-TOF MS in this study, are shown in the box. (TIF) [file pone.0275993.s004.TIF]
