# Supplementary material for: N-terminal peptide fragment constitutes core of amyloid deposition of serum amyloid A: An imaging mass spectrometry study
Source: PLoS One. 2022 Oct 14;17(10):e0275993. doi: 10.1371/journal.pone.0275993 (PMC9565386; doi:10.1371/journal.pone.0275993)
Supplement: S1 Table — (DOCX) [file pone.0275993.s005.docx]

**Supporting Table**

**S1 Table. Identification of peptides in cardiac tissues of patients with AA and ATTR amyloidosis**

| Amyloid type | *m/z* | *m/z* | *m/z* | *m/z* based on MS-Digest | |  |
| --- | --- | --- | --- | --- | --- | --- |
|  | IMS  TOF/TOF | IMS  Orbitrap | LC-MS  Orbitrap | mi | av | Ref. |
| ApoE | 1730.944 | N.D. | 1730.844 | 1730.84 | 1731.870 |  |
| VTN | 875.517 | 875.473 | 875.475 | 875.473 | 876.009 | [7] |
| VTN | 887.556 | 887.510 | N.D. | 887.510 | 888.064 | [6,7,8] |
| VTN | 1158.604 | 1158.588 | N.D. | 1158.623 | 1159.295 | [7] |
| VTN | 1314.829 | 1314.660 | 1314.682 | 1314.680 | 1315.481 | [6,7,8] |
| VTN | 1646.893 | N.D. | 1646.821 | 1646.817 | 1647.839 | [6,7,8] |
| VTN | 1666.908 | N.D. | 1666.781 | 1666.775 | 1667.855 | [6,7,8] |

The m/z detected by IMS TOF/TOF was confirmed by IMS orbitrap, LC-MS/MS, and MS-Digest (http://prospector.ucsf.edu/prospector/cgi-bin/msform.cgi?form=msdigest).

* LC-MS/MS data were obtained from a case of AA amyloidosis (see related data in reference ^3^; http://www.peptideatlas.org/PASS/PASS01558).

IMS, imaging mass spectrometry; LC-MS, liquid chromatography mass spectrometry; mi, monoisotopic molecular mass; av, average molecular mass; Ref, reference; SAA, serum amyloid A; TTR, transthyretin; SAP, serum amyloid P component; Apo A4, apolipoprotein A4; ApoE, apolipoprotein E; VTN, vitronectin.
